# Effect of PERLA®, a new cold-storage solution, on oxidative stress injury and early graft function in rat kidney transplantation model

**DOI:** 10.1186/s12882-024-03488-z

**Published:** 2024-02-22

**Authors:** Mohamed Bejaoui, Chérifa Slim, Carmen Peralta, Hassen Ben Abdennebi

**Affiliations:** 1 Laboratory of Human Genome and Multifactorial Diseases (LR12ES07), Faculty of Pharmacy, University of Monastir, Monastir, Tunisia; 2 Institut d’Investigacions Biomèdiques August Pi i Sunyer (IDIBAPS), 08036 Barcelona, Spain

**Keywords:** Oxidative stress, Preservation solutions, Kidney transplantation, Ischemia reperfusion injury

## Abstract

**Background:**

The composition of organ preservation solutions is crucial for maintaining graft integrity and early graft function after transplantation. The aim of this study is to compare new organ preservation solution PERLA® with the gold standard preservation solution University of Wisconsin (UW) regarding oxidative stress and early graft injury.

**Methods:**

In order to assess oxidative stress after cold storage, kidney grafts have been preserved for 18 h at 4° C in either UW solution or PERLA® solution and then assessed for oxidative stress injury (protocol 1). To assess kidney injuries and oxidative stress after reperfusion, rat kidneys were harvested, stored in cold UW or in PERLA® solutions for 18 h at 4 °C and then transplanted heterotopically for 6 h (protocol 2). PERLA® is a high Na^+^/low K^+^ solution including PEG-35 (1 g/L), trimetazidine (1 µM), carvedilol (10 µM) and tacrolimus (5 µM).

**Results:**

Our results showed that preservation of kidneys in PERLA® solution significantly attenuates oxidative stress parameters after cold storage and reperfusion. We found a significant decrease in oxidative damage indicators (MDA, CD and CP) and a significant increase in antioxidant indicators (GPx, GSH, CAT, SOD and PSH). Moreover, PERLA® solution decreased kidney injury after reperfusion (creatinine, LDH and uric acid).

**Conclusion:**

PERLA® solution was more effective than UW storage solution in preserving rat’s kidney grafts.

## Background

Ischemia reperfusion injuries (IRI) after transplantation remain an unresolved issue that exposes kidney grafts to short- and long-term complications [1, 2]. In order to reduce IRI, kidney grafts are submitted to hypothermic washout and storage (at 4 °C) with a cold storage solution that has a suitable composition to preserve the organs [3]. Hypothermia is deliberately used to reduce the oxygen need of cells and thus to decrease the metabolic activity of the graft. Consequently, the activity of many hydrolytic enzymes (phospholipases, proteases or endonucleases,…) is decreased and the destruction of the cell organelles and the structural macromolecules (cytoskeleton, proteins, nucleic acids,…) is prevented [4]. On the other hand, hypothermia activates several pathophysiological processes and multiple catabolic pathways that may impair graft functional recovery after reperfusion [5, 6]. Cold ischemia causes structural alterations, particularly in endothelial cells [5, 7]. Anaerobic metabolism prevailing during cold ischemia is characterized by a low level of adenosine triphosphate (ATP) production. Thus, all the ATPases proteins are inactivated, which entails intracellular acidosis and edema. In addition, Ca^2+^ is overloaded into cells due to decreased active Ca^2+^ efflux and reduced reuptake by the endoplasmic reticulum [8]. These changes are accompanied by mitochondrial dysfunction, which further impairs ATP production and strongly decreases protein synthesis, particularly antioxidant enzymes [4, 9]. As a result, cells become vulnerable to oxidative stress which leads to protein oxidation and ultimately cell death [10, 11].

Paradoxically, reperfusion of the grafts is also deleterious to cells. The restoration of blood flow during this phase suddenly reveals the shortcomings set up during the cold ischemia. Oxygen supply in the ischemic tissue leads to the generation of large amounts of reactive oxygen species (ROS) and an intense oxidative stress [2, 12]. Oxidative stress triggers a cascade of events that exacerbate cell death and tissue injury. These events constitute both a consequence and an amplification of damage mechanisms initiated during the cold ischemia phase.

The main goal of organ storage is to maintain the biochemical, morphological and functional integrity of the graft. A well-preserved graft should be able to restore, from the first minutes of reperfusion, its metabolic functions. This depends on several factors, including the composition of the solution used to preserve the organ [13, 14]. The University of Wisconsin (UW) solution has transformed the concept of solid organ preservation [3, 15]. Until now, this solution is the most used in transplant centers and it is considered as the gold standard solution for the preservation of the abdominal organs [16]. However, it has some disadvantages that limit its effectiveness. In fact, its hyper-potassium composition depolarizes the plasma membrane of endothelial cells leading to vasoconstriction, which increases vascular resistance and decreases blood flow in the graft during its reperfusion [17]. Another limitation is related to hydroxyethyl starch (HES), its oncotic support, which has been shown to increase the aggregation of red blood cells leading to inhomogeneous reperfusion of the grafts [18–21]. In addition, UW solution contains glutathione and allopurinol in order to reduce oxidative stress, however, their efficacy has never been clearly demonstrated [4].

The aim of the present study was to evaluate the effectiveness of a new cold storage solution called “PERLA” compared to UW solution. PERLA® composition was established on the basis of our understanding of the pathophysiology of IRI. It has an extracellular-like (high Na^+^/low K^+^) formulation and contains polyethylene glycol (PEG)-35, trimetazidine (TMZ), carvedilol and tacrolimus [22]. We compared the effect of PERLA® and UW solutions on oxidative stress after cold storage and after revascularization of renal grafts in a rodent heterotopic transplantation model.

## Methods

### Preservation solutions

UW solution is the Belzer UW solution (Bridge To Life, London, UK) in which dexamethasone, insulin and penicillin were omitted. PERLA® solution (Advanced Life Solutions, Bourg en Bresse, France) is a new preservation solution, patented by the university of Barcelona [22]. It has a high Na^+^/low K^+^ formulation including PEG-35 (1 g/L), and three stable, risk-free, and repositioned drugs for IRI protection: TMZ (1 µM), carvedilol (10 µM) and tacrolimus (5 µM). The compositions of UW and PERLA® solutions are shown in Table 1.

**Table 1:** Composition of belzer UW and PERLA preservation solutions

Components	Belzer UW	PERLA
Na^+^ (mM)	25	120
K^+^ (mM)	125	25
MgSO_4_ (mM)	5	5
H_2_PO_4_ (mM)	25	25
Lactobionic acid (mM)	100	100
Raffinose (mM)	30	30
Hydroxyethyl starch (g/l)	50	0
Polyethylene glycol 35 (g/l)	0	1
Adenosine (mM)	5	0
Allopurinol (mM)	1	0
Glutathione (mM)	3	0
Trimetazidine (µM)	0	1
Carvedilol (µM)	0	10
Tacrolimus (µM)	0	5

### Animals

Adult male Sprague Dawley rats weighing 200 to 250 g were used as donors and recipients. Animals were kept under standardized conditions (23 ± 2 °C and 12 h light-dark cycle) with ad libitum access to food and water until 24 h before surgery. The experiments were carried out in accordance with the European Union Regulations (Directive 2010/63/EU) for animal experiments and in accordance with ARRIVE guidelines for the reporting of animal experiments. Research protocol was approved by the Committee on Ethics in Life Sciences and Heath of the Higher Institute of Biotechnology of Monastir (CER-SVS/ISBM), University of Monastir, Tunisia.

### Protocol 1: Effect of UW and PERLA® on oxidative stress after cold storage

In a first study, both kidneys were harvested and randomly assigned into two groups (UW-1 and PERLA-1, *n* = 7). The kidneys were stored in cold UW or PERLA® solutions for 18 h at 4 °C. Then, tissue samples were collected and kept at 80 °C until evaluation of antioxidant enzymes activities and oxidative stress parameters. Small pellets of stone, stored in a -80 °C freezer, were employed for the rapid freezing of small tissue samples [23].

### Protocol 2: Effect of UW and PERLA® on oxidative stress after transplantation

In a second study, right rat kidneys were harvested, randomly stored in cold UW (UW-2 group, *n* = 7) or in PERLA® (PERLA-2 group, *n* = 7) solutions for 18 h at 4 °C and then transplanted heterotopically for 6 h. The results of these groups were compared with each other and, with those of a sham group (*n* = 4), where kidney pedicles were dissected off the surrounding peri-renal fat without performing cold storage or transplantation. At the end of the experiments, animals were sacrificed and blood samples and kidney tissues were sampled and immediately kept at -80 °C.

### Surgical procedures

We used an established transplanted rat kidney model of cold IRI [24]. All procedures were performed under isoflurane inhalation anesthesia (1.5% isoflurane at a rate of 0.8 L/min). Before starting surgery, normal volemia was maintained by iv infusion of 1 mL of saline. Warm saline was also instilled in the abdominal cavity during surgery. Rats were placed onto a heating pad to maintain body temperature at 37 ^∘^C.

The donor surgery and kidney harvesting were performed as follows: After a midline abdominal incision, the right kidney and its vascular pedicle were carefully dissected and exposed. The aortic collateral blood vessels around renal region (except right renal artery) were coagulated and then the ureter was cannulated. The aorta above the right renal artery was ligated and the right kidney was immediately washed with 5 mL of cold storage solution via a catheter inserted into the infrarenal aorta. This catheter was connected to a pressure transducer (Pression Monitor BP-1, Pression Instruments, Sarasota, FL) in order to control renal washing pressure which never exceeded 100 mmHg. Soon after, the kidney was excised and preserved in a small container of the corresponding cold storage solution (25 mL) at 4 °C for 18 h.

The recipient surgery and kidney implantation was performed as follow: The blood vessels were prepared to perform a heterotopic renal transplantation as previously described elsewhere [24]. The kidney graft was transplanted into the right iliac fossa by anastomosing end-to-side the donor renal artery and vein to recipient aorta and vena cava respectively using suture layers (CARDIO-PRENE 9/0). The graft ureter was connected to the bladder through a small cystostomy, and both recipients’ kidneys were then removed. Finally, the abdomen of rat recipients was closed using sutures and the animals were kept warm and allowed to recover.

### Determination of antioxidant enzymes activities

Renal tissues were homogenized in 0.1 M ice-cold potassium phosphate buffer (pH 7.4) to assess the activities of glutathione peroxidase (GPX), superoxide dismutase (SOD) and catalase (CAT).

Total superoxide dismutase (Cu-Zn SOD and Mn SOD) activity was determined by evaluating the ability of SOD to inhibit pyrogallol auto-oxidation according to the method of Marklund and Marklund [25]. Enzyme activity was measured at 420 nm and expressed as units/mg of protein. The activity of GPX and the concentration of the reduced glutathione (GSH) were measured by following the absorbance decrease at 412 nm of GSH in the presence of H_2_O_2_, at 25 °C, using the method of Flohé and Gunzler [26]. The activity of GPX was expressed as µmol of GSH oxidized/min/mg protein. The activity of CAT was assessed by measuring spectrophotometrically at 240 nm the decomposition rate of H_2_O_2_ using the method of Claiborne [27]. It was expressed as µmol H_2_O_2_ decomposed/min/mg of protein.

### Determination of oxidative stress parameters

Lipid peroxidation in renal tissues was evaluated by assessing malondialdehyde (MDA) and conjugated dienes (CD) formation. Tissues homogenization was carried out in ice-cold tris buffered saline (100 mM Tris, pH 7) or in distilled ice-water in order to assess MDA and CD concentrations, respectively. The MDA concentration was measured at 530 nm using the thiobarbituric acid assay [28]. The CD concentration was determined spectrophotometrically at 233 nm [29]. They were expressed as nmol/mg of protein.

Carbonyl protein (CP) groups were detected in renal tissue using Ellman’s reagent (10 mM DTNB prepared in 2.5 M HCl) according to the method of Levine et al. (Levine 1994). Samples were mixed with the DNPH (10 mM) for 1 h at room temperature. Then, they were centrifuged (4 000 g for 5 min at 4 °C) and the pellet was dissolved in 6.0 M of guanidine. CP content was measured spectrophotometrically at 370 nm. It was expressed as nmol/mg of protein.

Protein sulfhydryl (PSH) content was determined using the method of Seldak et al. [30]. Renal tissues were homogenized in 0.1 M ice-cold potassium phosphate buffer (pH 7.4). Then, total protein sulfhydryl groups (T-SH) and non-protein sulfhydryl groups (NP-SH) were assessed spectrophotometrically at 412 nm. The PSH level was measured by subtracting the NP-SH from T-SH. Result was expressed as mg/mg of protein.

Total protein concentration was determined by the Bradford assay.

### Determination of lactate dehydrogenase activity

Lactate dehydrogenase (LDH) in plasma was determined using commercial assay kit (Diagnostic System, Germany) according to manufacturer’s instructions. It was expressed as IU/L.

### Determination of kidney function

Creatinine and uric acid concentrations were measured in plasma using commercially available kits (Beckman Coulter, Inc., USA). They were expressed as µmol/L.

### Statistics

The data were presented as means ± SE. Comparisons between groups were made by unpaired Student’s t test for results of protocol 1, and by one-way analysis of variance (ANOVA) followed by the Tukey test for results of protocol 2 (Graph Pad Prism software 6.01). A p value < 0.05 was considered statistically significant.

## Results

### Protocol 1: Effect of UW and PERLA® on oxidative stress after the cold storage of kidneys

**Fig. 1:**
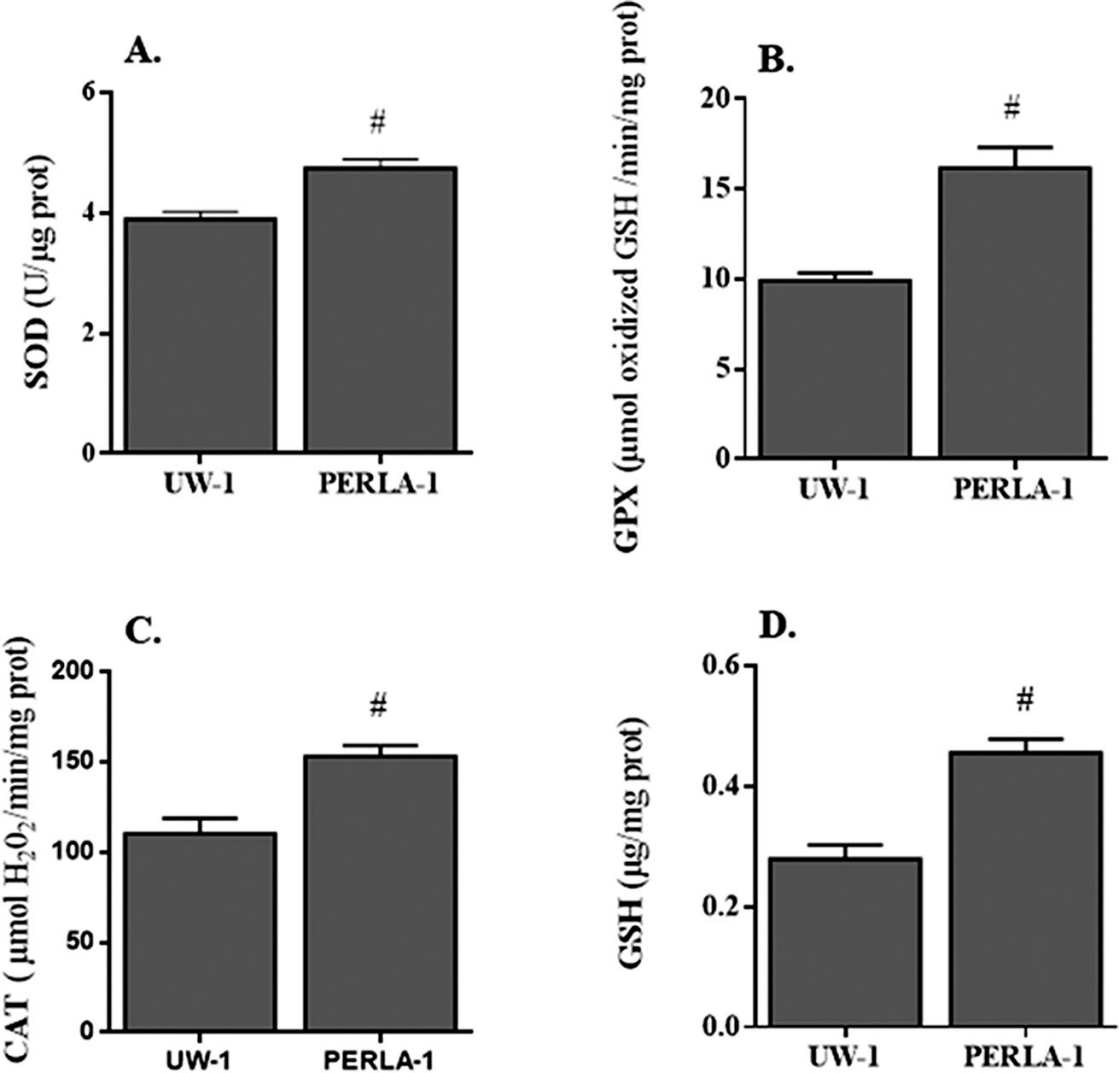
Activities of superoxide dismutase (**A**, SOD), glutathione peroxidase (**B**, GPX) and catalase (**C**, CAT) and concentration of reduced glutathione (**D**, GSH) in renal tissues. UW-1: kidney grafts were preserved in UW solution for 18 h; PERLA-1: kidney grafts were preserved in PERLA® solution for 18 h. # *P* < 0.05 versus UW

Antioxidant enzyme activities were significantly higher in rat kidneys preserved in PERLA® solution than those preserved in UW solution (Fig. 1). We found 3.9 ± 0.13 vs. 4.7 ± 0.14 IU/µg prot for SOD, 9.93 ± 0.43 vs. 16.18 ± 1.12 µmol oxidized GSH/min/mg prot for GPX, 110 ± 8.4 vs. 153 ± 6.0 µmol H_2_O_2_/min/mg prot for CAT and 0.28 ± 0.02 vs. 0.44 ± 0.02 µg/mg prot for GSH in kidney grafts preserved in UW and PERLA® solutions, respectively (*p* < 0.05).

**Fig. 2:**
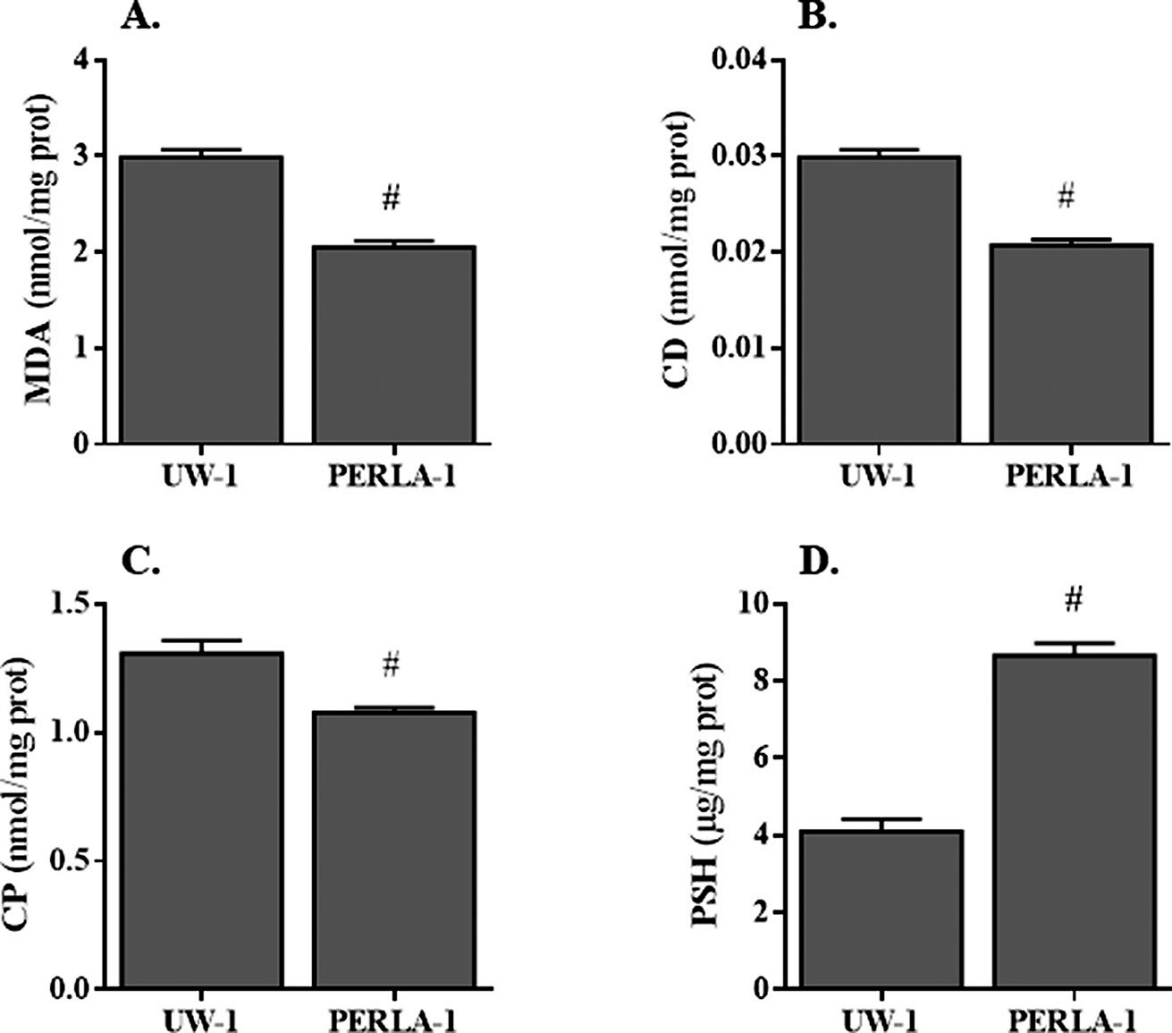
Concentrations of malondialdehyde (**A**, MDA), conjugated dienes (**B**, CD), carbonyl protein groups (**C**, CP) and protein sulfhydryl groups (**D**, PSH) in renal tissues. UW-1: kidney grafts were preserved in UW solution for 18 h; PERLA-1: kidney grafts were preserved in PERLA® solution for 18 h. # *P* < 0.05 versus UW-1

Concerning the oxidative stress damages and as compared with PERLA® solution (Fig. 2), renal preservation with UW solution increased the concentrations of MDA (3.0 ± 0.08 vs. 2.1 ± 0.06 nmol/mg prot in UW and PERLA® solutions, respectively, *p* < 0.05), CD (0.030 ± 0.001 vs. 0.021 ± 0.001 nmol/mg prot in UW and PERLA® solutions respectively, *p* < 0.05) and CP (1.3 ± 0.05 vs. 1.1 ± 0.02 nmol/mg prot in UW and PERLA® solutions respectively, *p* < 0.05) and decreased the content of PSH (4.1 ± 0.32 vs. 8.7 ± 0.31 µg/mg prot in UW and PERLA® solutions respectively, *p* < 0.05).

### Protocol 2: Effect of UW and PERLA® on acute reperfusion injury and oxidative stress after kidney transplantation

**Fig. 3:**
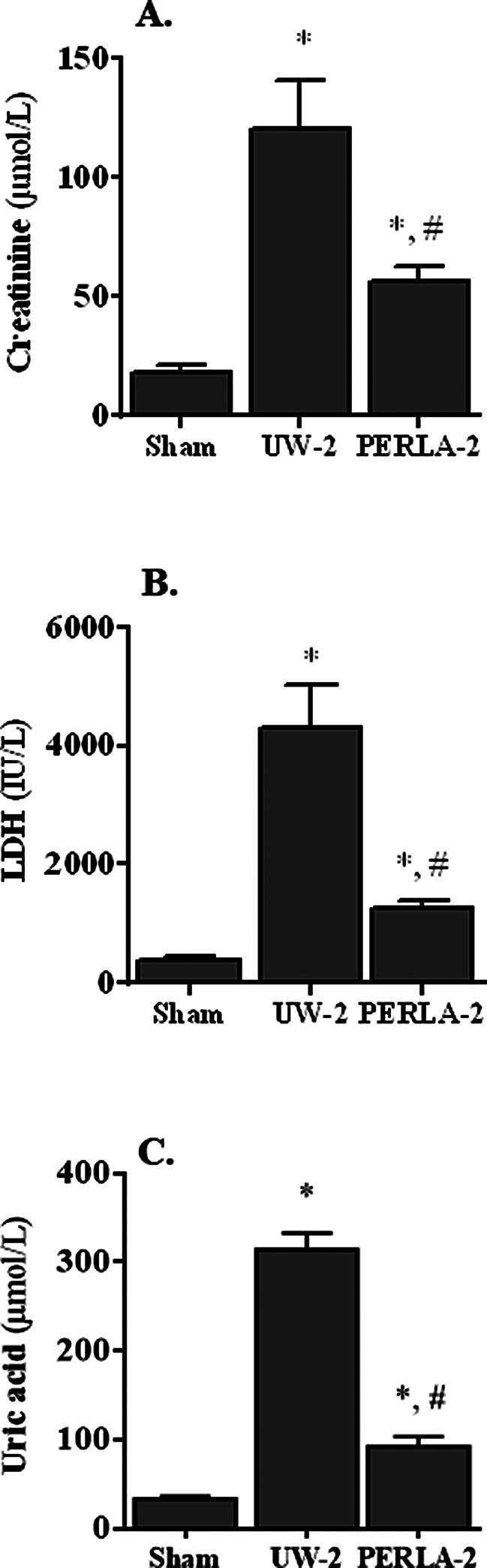
Concentration of creatinine (**A**), activity of lactate dehydrogenase (**B**, LDH) and concentration of uric acid (**C**) in plasma. Sham, kidney pedicles were only dissected off the surrounding peri-renal fat; UW-2: kidney grafts were preserved in UW solution for 18 h then vascularized for 6 h; PERLA-2: kidney grafts were preserved in PERLA® solution for 18 h then vascularized for 6 h. * *P* < 0.05 versus sham; # *P* < 0.05 versus UW-2

As compared to sham, kidney transplantation led to a significant increase in the oxidative stress and renal lesions parameters and, in parallel, to a decrease in the activities of all the antioxidant enzymes. Also, parameters of renal injuries were evaluated in the plasma of rats transplanted with kidneys preserved in UW or PERLA® solutions (Fig. 3). Results from the UW-2 group revealed high concentrations of creatinine and uric acid when compared to those found in the PERLA-2 group (120 ± 7.8 vs. 57 ± 6.0 µmol/L respectively for creatinine and 314 ± 18 vs. 93 ± 11 µmol/L respectively for uric acid, *p* < 0.5). Also, we noted that kidney preservation with PERLA® solution resulted in low LDH activity in comparison to that found in UW-2 group (1253 ± 151 vs. 4294 ± 745 U/L, respectively, *p* < 0.05).

**Fig. 4:**
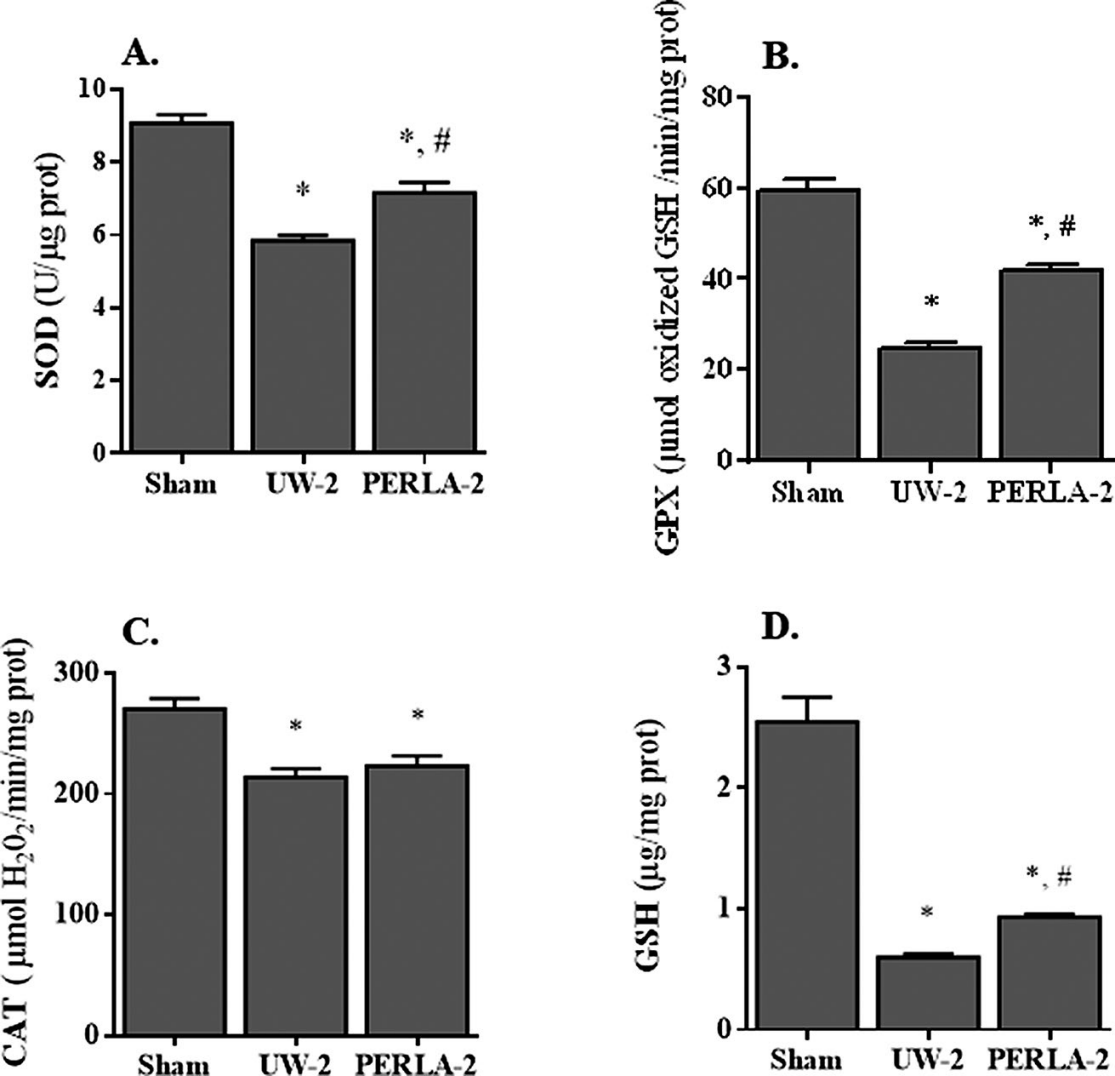
Activities of superoxide dismutase (**A**, SOD), glutathione peroxidase (**B**, GPX) and catalase (**C**, CAT) and concentration of reduced glutathione (**D**, GSH) in renal tissues. Sham, kidney pedicles were only dissected off the surrounding peri-renal fat; UW-2: kidney grafts were preserved in UW solution for 18 h then vascularized for 6 h; PERLA-2: kidney grafts were preserved in PERLA® solution for 18 h then vascularized for 6 h. * *P* < 0.05 versus sham; # *P* < 0.05 versus UW-2

**Fig. 5:**
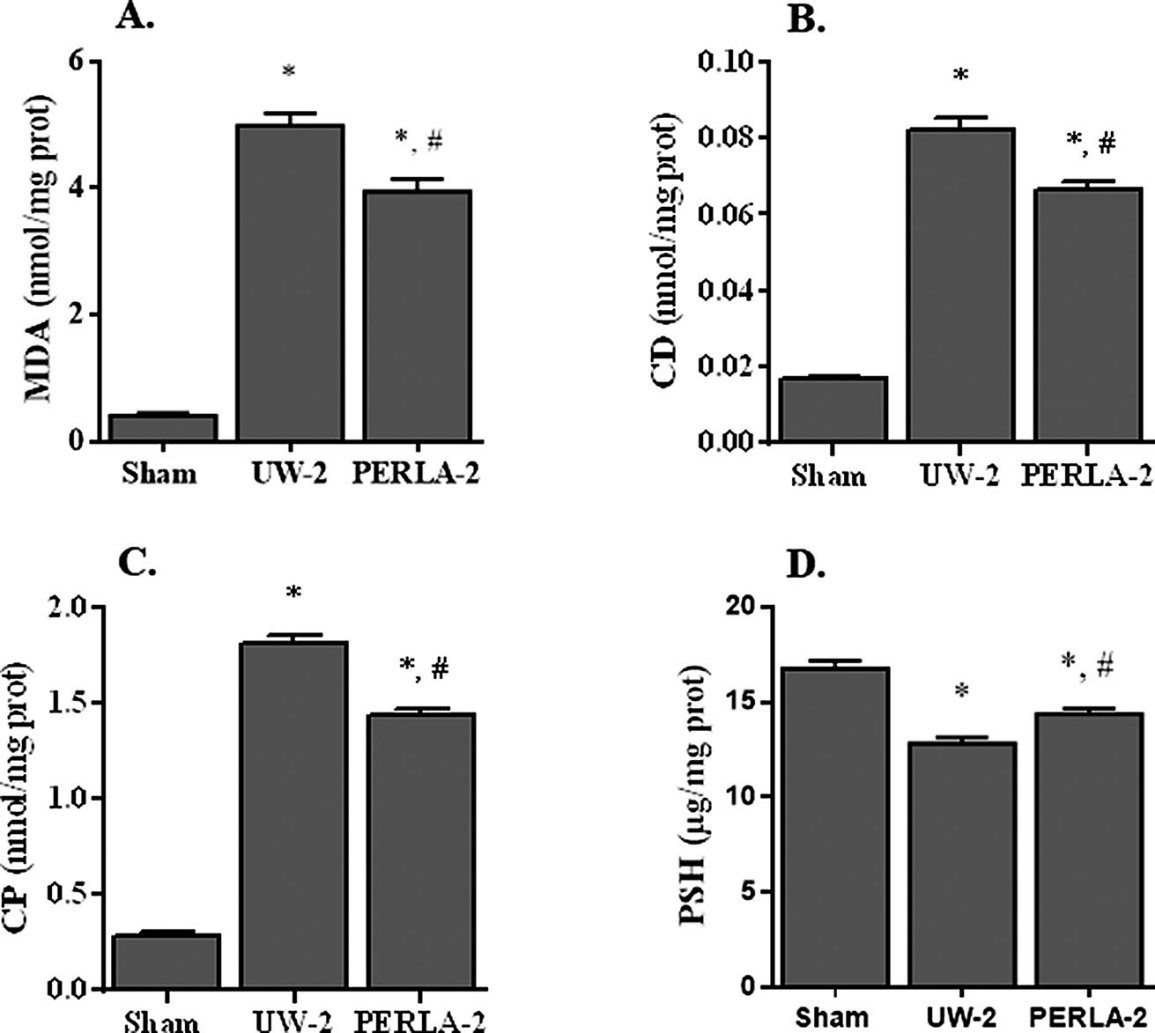
Concentrations of malondialdehyde (**A**, MDA), conjugated dienes (**B**, CD), carbonyl protein groups (**C**, CP) and protein sulfhydryl groups (**D**, PSH) in renal tissues. Sham, kidney pedicles were only dissected off the surrounding peri-renal fat; UW-2: kidney grafts were preserved in UW solution for 18 h then vascularized for 6 h; PERLA-2: kidney grafts were preserved in PERLA® solution for 18 h then vascularized for 6 h. * *P* < 0.05 versus sham; # *P* < 0.05 versus UW-2

Consistent with these observations, we observed that PERLA® solution better preserved antioxidant enzymes when compared to UW solution (Fig. 4). We noted respectively 7.16 ± 0.29 vs. 5.85 ± 0.14 U/µg prot for SOD (*p* < 0.05), 41.70 ± 1.59 vs. 24.75 ± 1.14 µmol oxidized GSH/min/mg prot for GPX (*p* < 0.05), 223.1 ± 8.6 vs. 213.8 ± 7.0 µmol H_2_O_2_/min/mg prot for CAT and 0.93 ± 0.02 vs. 0.60 ± 0.03 µg/mg prot for GSH (*p* < 0.05). In addition, the use of PERLA® solution markedly attenuated oxidative stress damage when compared to UW-2 group (Fig. 5). Indeed, we detected 3.95 ± 0.19 vs. 4.9 ± 0.19 nmol/mg prot for MDA, 0.067 ± 0.001 vs. 0.082 ± 0.003 nmol/mg prot for CD, 1.4 ± 0.03 vs. 1.8 ± 0.04 nmol/mg prot for CP and 14 ± 0.26 vs. 13 ± 0.30 µg/mg prot for PSH in PERLA-2 and UW-2 groups, respectively (*p* < 0.05).

## Discussion

In solid organ transplantation, the composition of the organ preservation solutions plays a key role in maintaining the quality of the grafts in order to restore normal metabolism upon reperfusion [1, 16, 31]. The storage solutions are also considered as the vectors of hypothermia to reduce the metabolic demands of the cells. However, hypothermia itself is responsible for cell injury and even worsens the damage caused by ischemia [3]. What is required from a cold storage solution is to be able to establish an appropriate physical environment able to limit cellular and interstitial edema and thus to reduce the alterations of cells structures. They must also provide a biochemical environment with appropriate electrolytes, metabolites and inhibitors to block the catabolic reactions responsible for irreversible damages [3].

ROS are the earliest and the most deleterious mediators produced throughout organ transplantation [2]. In the first protocol of our study, we compared UW and PERLA® solutions in oxidative stress after cold storage. Our results showed that PERLA® solution better preserved the antioxidant enzymes defenses than UW solution and we noticed less oxidative stress at the end of the kidney storage with PERLA® solution than with UW solution.

During cold ischemia, the blockage of the mitochondrial respiratory complexes leads to the accumulation of electrons, then to the increase in the electronegativity of the respiratory chain and the leakage of these electrons [32]. From the onset of the cold ischemia, the remaining available O_2_ is able to react with the free electrons and create the superoxide anion, a highly reactive and unstable ROS [33]. ROS are highly cytotoxic and can induce DNA damage, functional protein abnormalities, peroxidation of phospholipids and ultimately impaired function of organelles. Further, endothelial damage resulting from the action of ROS induces the loss of microvascular integrity [7, 34]. Cells are able to protect themselves against ROS actions. Endogenous enzymatic antioxidant systems, such as manganese-SOD (Mn-SOD), CAT and GPX may limit the effects of ROS [2]. However, hypothermia added to IRI strongly reduces the activity of these enzymes making the cells very sensitive to the attacks from ROS [35].

The restoration of blood flow, coupled with the massive supply of O_2_ to the graft after transplantation, triggers an exponential production of ROS [4]. As a consequence, ROS react with all the cellular macromolecules, leading to the destruction of cell membranes and instigating ferroptosis, an iron-driven cell death characterized by iron accumulation and excessive lipid peroxidation [2, 36]. The results of the second protocol showed that PERLA® solution was able to better maintain the activities of the antioxidant enzymes in the kidney transplants after their reperfusion than UW solution, which allowed them to be protected against oxidative stress damage. Therefore, renal function was improved when the kidneys were stored with PERLA® solution and the cytolysis was reduced.

The composition of UW and PERLA® solutions are rather different. The formulation of PERLA® was established based on our understanding of the pathophysiology of IRI, which has evolved well over the last few years. PERLA® has an extracellular (high Na^+^/low K^+^) washout base optimized by the oncotic contribution of PEG-35, and most importantly it includes, for the first time, novel IRI protective components: TMZ, carvedilol and tacrolimus. The effectiveness of PERLA® against oxidative stress would be due to the synergistic action of all these components.

Previous studies have shown that extracellular like preservation solutions decrease vascular constriction which allows a better graft washout [17]. In addition, PEG-35 in preservation solution can exert oncotic pressure thus limiting cellular and tissue edema [37, 38]. In fact, it has been shown that PEG-35-based UW solution was associated with the prevention of oxidative stress and the microcirculatory disorders in kidney transplantation [39]. The antioxidant effect of PEG-35 was related to its ability to maintain the integrity of the cell membrane and to prevent the mitochondrial swelling which is a source of ROS [39, 40].

In addition to its extracellular-like formulation and oncotic support, PERLA® solution includes three drugs, TMZ, carvedilol and tacrolimus, which effects against ischemia and oxidative stress have been well documented. TMZ (1-(2,3,4-trimethoxybenzyl) piperazine), is a cytoprotective agent which is found in the composition of PERLA® solution. Its beneficial effects have been explored in different experimental models of IRI [41–44]. Data indicate that TMZ preserves the integrity, metabolic functions and ionic permeability of mitochondria [45]. It improves microcirculation through activation of eNOS and NO production [46, 47]. TMZ is also recognized to have powerful antioxidant properties [44, 47, 48]. Faure et al. have revealed that the addition of TMZ to Euro-Collins or UW solutions improves kidney graft function in pigs [42, 43]. Ben Mosbah et al. have observed that the addition of TMZ to UW ameliorates the preservation of normal and steatotic livers [28]. They assumed that TMZ decreases oxidative stress, cytolysis and vascular resistance and preserves ATP content in cells. This effect could be related to the protection of mitochondria and the generation of NO by eNOS following the activation of AMPK [46, 47]. In addition, Pantazi et al. have demonstrated that TMZ exerts its protective effect on IRI associated with liver transplantation in rats, in part, by inducing the expression and activity of the sirtuin protein (SIRT)-1, which has among others antioxidant properties [49].

Carvedilol was incorporated into PERLA® solution owing to its multiple anti-ischemic properties. A study has already demonstrated a protective action of carvedilol when it is added to UW solution to preserve non-steatotic and steatotic livers of rats [50]. Indeed, the authors found an improvement in cytolysis, vascular resistance and oxidative stress parameters after the use of carvedilol. In parallel, they observed a preservation of the integrity of mitochondria and in the content of ATP in tissues. However, the main anti-ischemic properties of carvedilol have been described in warm ischemia. Under these conditions, numerous studies strongly suggested that carvedilol can exert its antioxidant effects thanks to its capacity to scavenge O_2_.^−^ and HO^−^ anions and to sustain the activity of endogenous antioxidant systems [51, 52]. An ability to prevent ferroptosis has been also attributed to carvedilol [53]. The antiferroptotic effect was linked to its iron-chelating and lipid peroxyl radical–scavenging activities [53–55].

PERLA® solution contains tacrolimus which has been shown to decrease oxidative stress and improve IRI [56–59]. These studies have shown that treatment with tacrolimus preserves cellular ATP content, lowers the production of ROS and maintains the activities of antioxidant enzymes. In addition, tacrolimus has been found to preserve mitochondria and to protect the electron transport chains and the mPTP opening [60, 61].

In our study, we conducted a comparison between PERLA® and the UW solution to evaluate their effects on oxidative stress and early graft injury during a 6 h reperfusion period. The results showed that PERLA® exhibited significant benefits in reducing oxidative stress and kidney injury. The decision to use a short time frame was made to obtain preliminary insights into the impact of the preservation solution on oxidative stress. It should be noted that the peak levels of plasma creatinine typically occurs between 6 and 18 h after transplantation [34]. However, it is important to exercise caution when extending these results to longer preservation times for clinical settings.

The translational potential of PERLA® as an organ preservation solution necessitates a comprehensive assessment of potential side effects and safety considerations. This is particularly crucial given the inclusion of TMZ, carvedilol, and tacrolimus in PERLA®, which are commonly utilized in clinical settings but are being repositioned here for organ preservation. While the beta-blocking properties of carvedilol raise concerns regarding heart rate and blood pressure regulation [62], and tacrolimus has the potential for nephrotoxicity [63], it is noteworthy that both carvedilol and tacrolimus in PERLA® are administered in low concentrations with a relatively short exposure time. Moreover, prior to reperfusion, the grafts undergo thorough washing to eliminate any residual solution components, further minimizing the risk of these substances affecting the recipient.

## Conclusion

**Fig. 6:**
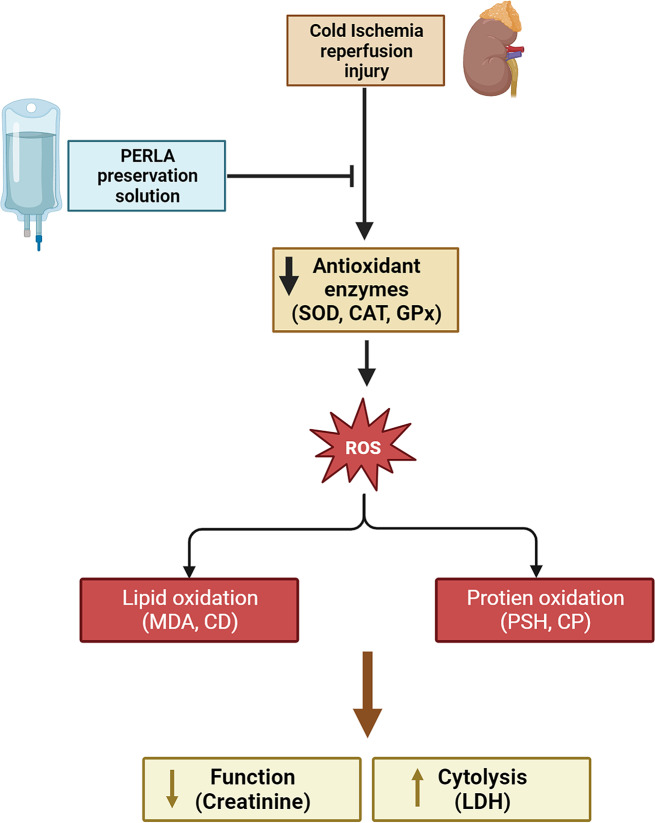
Effect of PERLA® organ preservation solution on oxidative stress and rat kidney function. PERLA® effectively decreased oxidative stress by enhancing antioxidant enzymes (SOD, CAT, GPx), resulting in reduced lipid peroxidation (MDA, CD) and protein oxidation (CP, PSH). This led to a decrease in cytolysis (LDH) and the preservation of kidney function, as evidenced by lower creatinine levels. (SOD - Superoxide Dismutase, CAT - Catalase, GPx - Glutathione Peroxidase, MDA - Malondialdehyde, CD: Conjugated dienes, CP: Carbonyl Protein, PSH: Protein Sulfhydryl, LDH: Lactate Dehydrogenase)

In conclusion, our study shows that PERLA® solution was more effective in preserving rat’s kidney grafts than the gold standard UW solution. PERLA® solution was able to reduce oxidative stress and to improve the functional recovery of the transplanted kidneys (Fig. 6). The benefit of PERLA® solution may be explained by its composition containing TMZ, carvedilol and tacrolimus, which are multi-target drugs and clinically well-known compounds. Further research, including extended evaluation periods and clinical trials, is warranted to fully elucidate the long-term impact of PERLA® and establish its place in improving organ preservation strategies for successful transplantation.

## Data Availability

Data generated or analyzed during this study are provided in full within the published article.

## Abbreviations

UW: University of Wisconsin
IRI: Ischemia reperfusion injuries
ATP: Adenosine triphosphate
PEG: Polyethylene glycol
GPX: Glutathione peroxidase
SOD: Superoxide dismutase
CAT: Catalase
ROS: Reactive oxygen species
TMZ: Trimetazidine
GSH: Reduced glutathione
MDA: Malondialdehyde
CD: Conjugated dienes
CP: Carbonyl protein
PSH: Protein sulfhydryl
